# An In Vitro Study Evaluating the Safety of Mesalazine on Human Nasoepithelial Cells

**DOI:** 10.3390/ijms25052796

**Published:** 2024-02-28

**Authors:** William Murphy, Sha Liu, Shari Javadiyan, Erich Vyskocil, Sholeh Feizi, Claudio Callejas, Peter-John Wormald, Sarah Vreugde, Alkis J. Psaltis

**Affiliations:** 1 Department of Surgery-Otolaryngology Head and Neck Surgery, Basil Hetzel Institute for Translational Health Research, Central Adelaide Local Health Network, Adelaide 5011, Australia; william.murphy@adelaide.edu.au (W.M.);; 2 The Department of Surgery, Faculty of Health and Medical Sciences, University of Adelaide, Adelaide 5000, Australia; 3 Department of Otolaryngology, Head and Neck Surgery, Medical University of Vienna, 1090 Vienna, Austria; 4 Department of Otolaryngology, Head and Neck Surgery, The Ohio State University, Columbus, OH 43210, USA; 5 Department of Otolaryngology, Pontificia Universidad Católica de Chile, Santiago 8320165, Chile

**Keywords:** chronic rhinosinusitis, IL-6, inflammation, mesalazine, TNF-α

## Abstract

Chronic rhinosinusitis (CRS) is a disease characterised by the inflammation of the nasal and paranasal cavities. It is a widespread condition with considerable morbidity for patients. Current treatment for chronic rhinosinusitis consists of appropriate medical therapy followed by surgery in medically resistant patients. Although oral steroids are effective, they are associated with significant morbidity, and disease recurrence is common when discontinued. The development of additional steroid sparing therapies is therefore needed. Mesalazine is a commonly used therapeutic in inflammatory bowel disease, which shares a similar disease profile with chronic rhinosinusitis. This exploratory in vitro study aims to investigate whether mesalazine could be repurposed to a nasal wash, which is safe on human nasoepithelial cells, and retains its anti-inflammatory effects. CRS patients’ human nasal epithelial cells (HNECs) were collected. HNECs were grown at an air-liquid interface (ALIs) and in a monolayer and challenged with mesalazine or a non-medicated control. Transepithelial electrical resistance, paracellular permeability, and toxicity were measured to assess epithelial integrity and safety. The anti-inflammatory effects of mesalazine on the release of interleukin (IL)-6 and tumour necrosis factor alpha (TNF-α) were analysed using human leukemia monocytic cell line (THP-1). mesalazine did not impact the barrier function of HNEC-ALIs and was not toxic when applied to HNECs or THP-1 cells at concentrations up to 20 mM. mesalazine at 0.5 and 1 mM concentrations significantly inhibited TNF-α release by THP-1 cells. mesalazine effectively decreases TNF-α secretion from THP-1 cells, indicating the possibility of its anti-inflammatory properties. The safety profile of mesalazine at doses up to 20 mM suggests that it is safe when applied topically on HNECs.

## 1. Introduction

Chronic rhinosinusitis (CRS) is defined as chronic paranasal sinus mucosal inflammation [1] lasting over 12 weeks with characteristic clinical manifestations. It affects around 10% of Western populations, making it an extremely prevalent disease [2,3,4]. CRS causes nasal obstruction, discharge, facial pain, headache, and an altered or impaired sense of smell and taste [5]. Patients require long-term monitoring and intensive care, and suffer a substantially diminished quality of life [6]. Antibiotics, corticosteroids, and saline nasal irrigations are commonly used to release the symptoms of CRS. These treatments can be ineffective in some cases, whilst the emergency of antibiotic resistance can hinder the efficacy of antibiotics in a clinical setting [7,8]. In such instances, surgical intervention is required [5]. Surgical procedures may alleviate symptoms and facilitate disease control, although disease recurrence cannot be prevented [9].

Chronic sinonasal inflammation involves the secretion of numerous inflammatory markers and can be associated with the development of nasal polyps [10,11]. Eosinophilic inflammation commonly characterizes the inflammatory response associated with CRS and has been well studied. Recently, the role of macrophages in the inflammatory process has gained increasing interest. Following an infection, macrophages in peripheral tissue (such as the sinonasal mucosa) function as immune system sentries and produce interleukin (IL)-1β, tumour necrosis factor (TNF)-α, and interleukin (IL)-6 [12]. The secretion of these cytokines is elevated in the context of CRS [13,14]. CRS is also associated with an increase in CD16+ monocytes, which then further differentiate into macrophages [15]. This has been supported by the presence of macrophage infiltration in CRS [16,17]. The monocyte-derived macrophages in sinonasal tissue can initiate Th-2 responses associated with CRS [16,18].

Mesalazine, also known as mesalamine or 5-aminosalicylic acid (5-ASA), is used to treat inflammatory bowel illnesses such as Crohn’s disease and ulcerative colitis [19]. Oral and topical (enema, foam, and suppositories) mesalazine has been shown to be effective for treating active ulcerative colitis and maintaining remission [20]. mesalazine’s anti-inflammatory therapeutic efficacy is widely acknowledged in the treatment of inflammatory bowel disease. Recent research suggests that the anti-inflammatory effects of 5-ASA are mediated at least in part by a reduction in the production of macrophage-derived cytokines such as TNF-α, Interleukin-1 beta (IL-1β), and Interleukin-6 (IL-6) [21,22,23]. mesalazine, in general, is well tolerated and considered safe for long-term use [24,25]. However, its pharmacological processes and mode of action are insufficiently understood [19].

Inflammatory bowel disease and certain CRS endotypes share many similar histological features, including neutrophil and plasma cell infiltrates, papillary hyperplasia, basement membrane thickening, oedema, ulcer formation, polypoid formation, and, for some end stage patients, fibrosis and scar formation [26]. Although it is known that mesalazine has anti-inflammatory activities and a favourable safety profile in the digestive system, its potential anti-inflammatory effect and safety on the sinonasal mucosa have not yet been studied.

THP-1 cells are a monocytic cell line used to investigate the functions, activities, signaling pathways, nutrition, and drug transport of monocytes and macrophages [27]. Therefore, in this study, the safety of various dosages of mesalazine was investigated on in vitro cultured human nasal epithelial cells (HNECs), as well as the anti-inflammatory effects on THP-1 cells, which are used to study monocyte/macrophage-related processes.

## 2. Results

### 2.1. Mesalazine Does Not Appear to Affect the Mucosal Barrier Function of Primary Human Nasal Epithelial Cell Cultures

**Figure 1:**
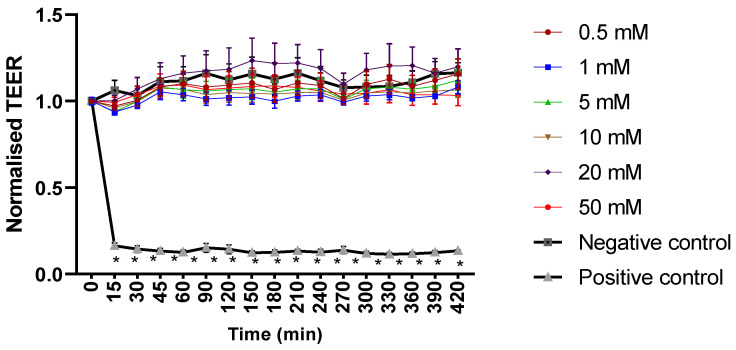
Mesalazine has no significant effect on HNEC-ALI TEER. HNEC-ALI TEER measurement after exposure to different concentrations of mesalazine (0.5, 1, 5, 10, 20 and 50 mM). ALI complete media and 10% Triton X-100 served as a negative and positive control, respectively. Statistical significance was determined by comparing each treatment condition to the negative control using one-way ANOVA. The values are shown in mean ± SD for *n* = 3. TEER was normalised against the value at time 0. *: *p* < 0.05. HNEC-ALI: human nasal epithelial cell-air liquid interface; TEER: transepithelial electrical resistance; SD: standard deviation.

**Figure 2:**
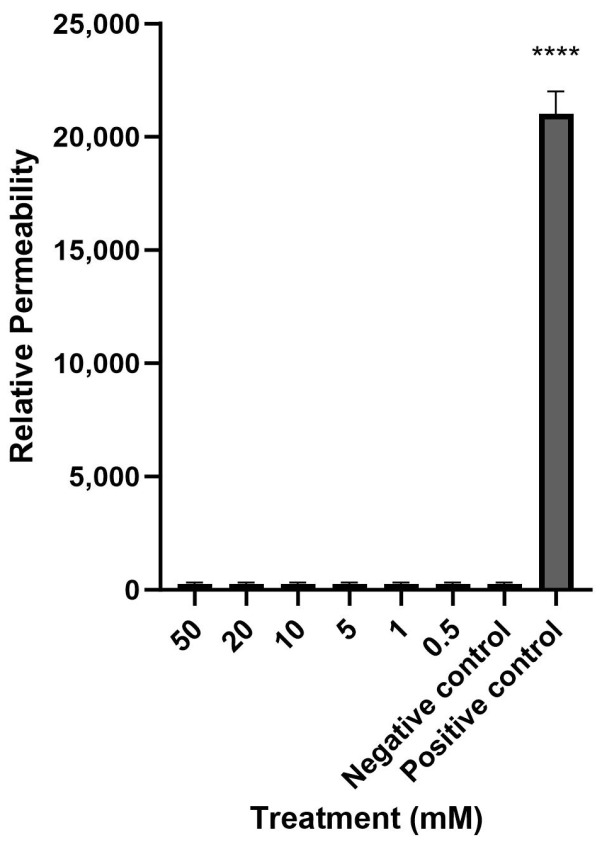
Mesalazine has no significant effect on HNEC-ALI paracellular permeability. The paracellular permeability was measured by the FITC-Dextran Assay at 7 h, following exposure to different concentrations of mesalazine. ALI complete media and 10% Triton X-100 served as the negative and positive controls, respectively. The significance was determined by comparing different treatment groups with the negative control. Experiments were performed in triplicates. ****: *p* < 0.0001. HNEC-ALI: human nasal epithelial cell-air liquid interface; FITC: fluorescein isothiocyanate.

Mesalazine did not alter the Trans-Epithelial Electrical Resistance (TEER) of the HNEC-ALI cultures at all tested concentrations (0.5, 1, 10, 20 and 50 mM) at each tested time point (up to 7 h), compared to the negative control (Figure 1). Furthermore, mesalazine applied for 7 h did not appear to have a significant effect on the paracellular permeability of the HNEC-ALI cultures at any of the concentrations tested (Figure 2).

### 2.2. Mesalazine Does Not Appear to Be Cytotoxic to HNECs in Concentrations < 50 mM

**Figure 3:**
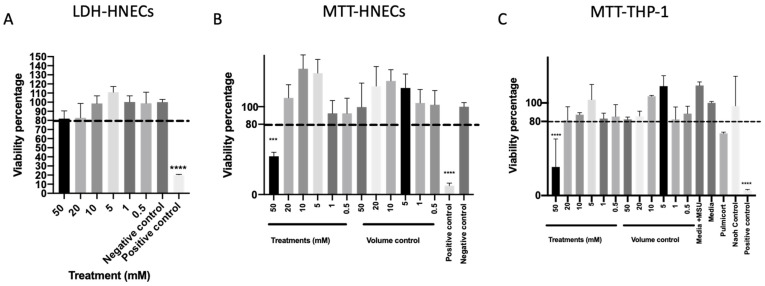
Mesalazine appears to be non-toxic to HNECs and THP-1 Cells in concentrations < 50 mM. (**A**) The LDH of HNEC-ALI cultures after 7 h of treatment with various concentrations of mesalazine. (**B**) MTT assay of HNECs monolayers and (**C**) THP-1 cell viability were measured after treatment with various concentrations of mesalazine for 24 h. Experiments were performed with three replicates. The significance was determined by comparing it with the negative control. ***: *p* < 0.001, ****: *p* < 0.0001. Negative control = ALI complete media; Positive control = 10% Triton X-100; Volume control = saline 0.9% in media. LDH: Lactate Dehydrogenase; HNEC-ALI: human nasal epithelial cell-air liquid interface; 3-(4,5-Dimethylthiazol-2-yl)-2,5-Diphenyltetrazolium Bromide: MTT.

To further evaluate the safety of mesalazine, cell cytotoxicity was measured after 7 h of Mesalazine exposure to HNEC-ALI cultures. None of the tested concentrations of mesalazine-induced lactate dehydrogenase (LDH) release compared to the negative control (Figure 3A). Furthermore, mesalazine at concentrations of up to 20 mM applied for 24 h to HNECs (Figure 3B) or THP-1 cell cultures (Figure 3C) did not affect cell viability as measured by nicotinamide adenine dinucleotide phosphate (NAD(P)H)-dependent cellular oxidoreductase enzymes activity (3-(4,5-Dimethylthiazol-2-yl)-2,5-Diphenyltetrazolium Bromide (MTT) assay) compared to any of the tested controls. However, 50 mM mesalazine reduced the cell viability below 80% for both cell types in the MTT assay (*p* < 0.0001) (Figure 3B,C) this was not seen at any of the lower concentrations.

### 2.3. Mesalazine Reduces the Release of TNF-α by THP-1 Cells

**Figure 4:**
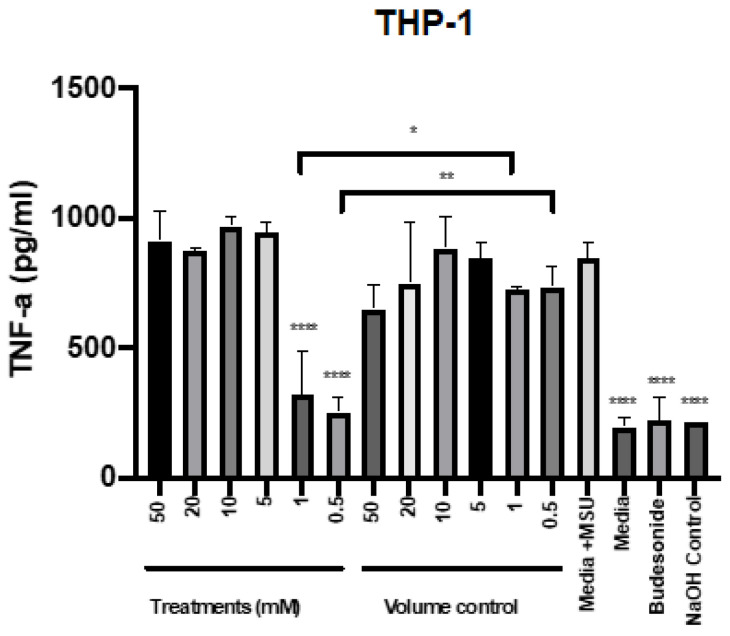
Mesalazine has anti-inflammatory effects on the production of TNF-α by THP-1 cells. The TNF-α production after 7 h of treatment with various concentrations of mesalazine on THP-1 cells. The significance was determined by comparing it with the control. *: *p* < 0.02, **: *p* < 0.01, ****: *p* < 0.0001. The experiments were performed three times. Negative controls = media; Volume control = Saline 0.9% in media and positive treatment control = budesonide; Control = NaOH. TNF-α: Tumour Necrosis Factor-alpha; NaOH: sodium hydroxide. MSU: Monosodium Urate.

To determine the potential of mesalazine to reduce TNF-α, the different concentrations were applied to THP-1 cells in the presence of the proinflammatory agent monosodium urate (MSU). Budesonide was used as an anti-inflammatory standard of care control. The production of TNF-α after stimulation with MSU at different time points was assessed for THP-1 cells (Supplementary Figure S1). Stimulation with MSU for 6 h significantly induced TNF-α release by THP-1 cells. Compared with this positive control, the addition of 0.5 and 1 mM mesalazine as well as the standard of care therapy (budesonide) significantly reduced TNF-α production by MSU-stimulated THP-1 cells to baseline level. In contrast, higher concentrations of mesalazine (from 5 mM up to 50 mM) did not reduce TNF-α release compared to the positive control (Figure 4).

### 2.4. Mesalazine Has No Effects on the Release of IL-6 by HNEC-ALI Cultures

**Figure 5:**
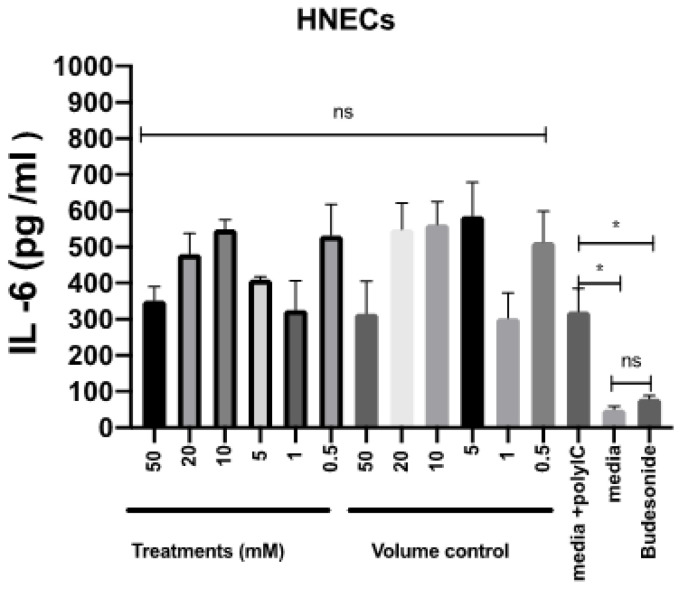
Mesalazine has no effects on the release of IL-6 by HNEC-ALI cultures. The IL-6 production after stimulation with poly (I:C) and 7 h of treatment with various concentrations of mesalazine or volume control in HNEC-ALI cultures. Budesonide was used as a standard of care control. The experiments were performed with three replicates. Negative control = Ex plus complete media; Positive control = media plus poly (I:C); Volume control = Saline 0.9% in media and Positive treatment control = Budesonide. *: *p* < 0.02, ns = not significant. IL-6: interlukin-6; HNEC-ALI: human nasal epithelial cell-air liquid interface.

To assess the potential for anti-inflammatory effects of mesalazine, the release of IL-6 by HNEC-ALI cultures was measured after stimulation with polycytidylic acid poly (I:C) for 7 h in the presence or absence of various concentrations of mesalazine. Budesonide was used as an anti-inflammatory standard of care control. Compared with the positive control, mesalazine did not show any alteration in IL-6 production by HNECs. In contrast, there was a significant reduction in IL-6 release in cells treated with budesonide, compared to the positive control (*p* < 0.05), reducing IL-6 levels to baseline (Figure 5).

## 3. Discussion

In this study, we evaluated the in vitro safety and potential for the anti-inflammatory effects of mesalazine in CRS patient-derived HNEC-ALI cultures and THP-1 monocytes/macrophages, respectively. Our findings revealed that mesalazine did not affect the barrier function of HNEC-ALIs and exhibited no cytotoxicity on either HNECs or THP-1 cells at concentrations up to 20 mM. Furthermore, mesalazine displayed effectiveness at lower concentrations (0.5 and 1 mM) in reducing TNF-α release by THP-1 cells. However, higher concentrations (5 mM to 50 mM) did not achieve the same reduction. This discrepancy is likely due to the necessity for adequate dissolution of mesalazine in solution to optimize receptor contact and impact the target tissue. With documented solubility in water < 1 mg/mL, the lower concentration (1 mM) at 0.1532 mg/mL aligns with this range. Conversely, the higher concentration (20 mM) at 3.06 mg/mL is likely insufficiently dissolved, possibly containing undissolved particles, thereby limiting mesalazine’s efficacy on its target. The current treatment for chronic rhinosinusitis routinely comprises intranasal corticosteroids and antibiotics in cases of infectious exacerbations. Short courses of oral corticosteroids are also given in some cases. If these therapies fail, patients are offered surgical intervention [5]. Therapy choices for chronic rhinosinusitis are therefore limited, and current disease management is often unsuccessful [9].

CRS has recently been attempted to be subclassified into inflammatory endotypes [5]. The notion is that a variety of inflammatory pathways contribute to a defective interaction at the sinonasal mucosa between the host and the environment [5]. Three inflammatory endotypes are implicated in CRS, which is characterized by Th1, Th2, and Th17 cells.

Type 2 inflammation is dominated by eosinophilic-driven inflammation and is linked to chronic inflammatory illnesses, including asthma. Previous studies on type 2 inflammation have been conducted in murine mammals using induction with poly (I:C). [28,29]. Whilst type 1 inflammation is driven by interferon-gamma (IFN-*γ*) and TNF-α and is associated with a neutrophilic/macrophage-driven inflammation [30], toll-like receptor 2 (TLR2) is exhibited at a greater level in CRS patients, particularly in Caucasian populations. The stimulation of TLR2 activates the myeloid differentiation primary response 88 (MYD88)-dependent signalling cascade, and the nuclear factor kappa B (NF-kappa B) pathway resulting in a large production of cytokines and chemokines such as TNF-α and IL-6 [31]. It has been demonstrated that TNF-α is a pro-inflammatory cytokine involved in chronic rhinosinusitis [13,14]. Macrophages play a role in TNF-α production and are also elevated locally and peripherally in chronic rhinosinusitis [13,14]. The induction of the NF-kappa B pathway in murine mammals has been achieved using MSU [32]. MSU triggers macrophage-induced inflammation by translocating the p65 subunit in the NF-kappa B pathway, a pathway implicated in CRS [6,7]. TNF-α, a major cytokine produced by macrophages through NF-kappa B mediation [8], is induced by MSU in THP-1 cells [9]. While MSU is not directly linked to CRS, it serves as an in vitro tool to study the NF-kappa B pathway and its effects on cytokines are relevant to CRS.

Aminosalicylates are a group of medications such as sulfasalazine, mesalazine, and mesalamine which have been used to reduce the inflammation of the intestine in Crohn’s disease [32]. It is reported that sulfasalazine has a lower effect on the remission of Crohn’s disease in comparison to both corticosteroids and placebos [33]. Furthermore, placebo treatment is also more effective in the remission of Crohn’s disease rather than mesalamine at higher doses of 3.2 to 4 g/day [33]. Mesalamine at a high dose (4 to 4.5 g/day) has a lower influence on the control of Crohn’s disease in comparison to budesonide [33]. Sulfasalazine (alone or in combination with corticosteroids) is superior to mesalamine at induction of Cronh’s disease remission [33]. Mesalazine has been employed as a treatment for inflammatory bowel disease for some time. Mesalazine’s mechanism of action is currently unknown; however, it is believed to entail the suppression of the NF-kappa B pathway and reduction of TNF-α production [21,22,23]. The obvious question that arises then is whether mesalazine could potentially have an application in certain types of CRS by inhibiting inflammation driven through this pathway. Although comparing the dosage of mesalazine in our study with that used in Crohn’s disease treatment is challenging due to the drug’s rapid metabolism within the bowel, mesalazine undergoes swift metabolism by intestinal N-acetyltransferase 1 (NAT1) to an inactive form, N-acetyl-5ASA [34]. Since the nasal mucosa lacks NAT1, a direct comparison would be limited, as this metabolism alters the required dosage. Additionally, given the considerable length of the small intestine (9–16 feet) and the multifocal nature of Crohn’s disease, significant mesalazine metabolism may occur before reaching the affected sites.

As mesalazine has never been administered topically in CRS patients, we evaluated the safety and efficacy of mesalazine’s anti-inflammatory properties in an in vitro setting for the first time. Mesalazine did not significantly alter the TEER during 7 h at concentrations up to 50 mM and did not alter the paracellular permeability, indicating that it does not influence the integrity of the mucosal barrier. Up to 50 mM, there was no significant effect on LDH levels; however, 50 mM mesalazine did reduce cell viability of both HNECs and THP-1 cells in the MTT assay. This indicates mitochondrial malfunction and potential toxicity at this higher dose of mesalazine. However, the fact that 20 mM and lower concentrations did not reduce cell viability suggests that this toxicity is dose dependent. Furthermore, and interestingly, given that the strongest anti-inflammatory effect was seen at lower concentrations of 1 mM and 0.5 mM, this implies a good in vitro safety profile of mesalazine at concentrations that hold promise for their anti-inflammatory effects in the context of CRS. A limitation of this study is that the anti-inflammatory effects were studied on in vitro THP-1, as macrophages harvested from the nasoepithelium cannot replicate and therefore cannot be cultured with the HNECs. Further, in vivo studies will need to be conducted to validate these promising in vitro safety and effectiveness data.

IL-6 is a cytokine produced through the NF-Kappa B pathway [35]. Intriguingly IL-6 showed no significant change when mesalazine was applied in all concentrations up to 50 mM on HNECs in our study. However, mesalazine at doses of 0.5 mM and 1 mM dramatically decreased TNF-α production by THP-1 monocytes/macrophages, whereas TNF-α production was inconsistent by HNECs. This supports the notion that mesalazine possesses anti-inflammatory effects that include TNF-α in monocytes/macrophages. In our studies, TNF-α could not be consistently induced in HNEC cultures. This has also been observed in other studies where the secretion of TNF-α by HNECs is variable and inconsistent [36]. Patients’ sinus tissue, in particular CRS tissue, is known to be enriched in macrophages and those cells are thought to play an important role in the pathophysiology of this disease [16,17]. Therefore, this study supports the potential for mesalazine to be used as a topical therapy to reduce inflammation in CRS patients. Given the potent effects of low concentrations of mesalazine seen on reducing the pro-inflammatory TNF-α levels produced by THP-1 cells, further in vivo studies are warranted to validate these findings towards clinical translation.

## 4. Materials and Methods

### 4.1. Mesalazine Preparation

Mesalazine solution was prepared by dissolving powdered mesalazine (>99.9% purity) (Sigma Aldrich, St. Louis, MO, USA) in sterilised Mili Q water and adjusted to pH to 7 using 1 mM sodium hydroxide (NaOH) solution. The mesalazine solution was then diluted into either PneumaCult™-Ex Plus Basal Medium (STEMCELL Technologies, Tullamarine, VIC, Australia), PneumaCult™-Ex Plus 50X Supplement (STEMCELL Technologies, Tullamarine, VIC, Australia), and penicillin-streptomycin (Thermo Scientific, Walthman, MA, USA) (designated as Ex Plus complete media)] or PneumaCult™-ALI Basal Medium (STEMCELL Technologies, Tullamarine, VIC, Australia); PneumaCult™-ALI 10X Supplement; penicillin-streptomycin/amphotericin B (Thermo Scientific, Waltham, MA, USA); And PneumaCult™-ALI Maintenance Supplement (STEMCELL, Vancouver, BC, Canada) (ALI complete media)] to achieve the final concentrations of 0.5 mM, 1 mM, 10 mM, 20 mM and 50 mM. The mesalazine solution was covered with foil paper in all experiments unless specified.

### 4.2. Human Ethics Approval and Participant Recruitment

Ethics approval for the harvesting of nasal brushings was granted by The Central Adelaide Local Health Network Human Research Ethics Committee (reference HREC/15/TQEH/132). Patients undergoing endoscopic sinus surgery (ESS) for chronic rhinosinusitis were recruited for the study. The diagnostic criteria used for CRS were in accordance with the American Academy of Otolaryngology and Head and Neck Surgery and the European Position Statement [5]. All patients provided written informed consent before the study initiation. The samples were de-identified and coded before use. The exclusion criteria included: (1) age below 18; (2) pregnancy; (3) active smokers for more than 3 months before the recruitment; (4) corticosteroid or antibiotic usage 4-weeks before the recruitment; and (5) systemic diseases, including cancer, hepatic and renal failure, and other conditions causing immunosuppression or for which immunosuppressant medication was part of their treatment.

### 4.3. Harvesting and Culturing HNECs In Vitro

Primary HNECs from patients with chronic rhinosinusitis were harvested from nasal polyp mucosa by gentle brushing [37]. The extracted cells were suspended in Ex Plus complete media (STEMCELL Technologies, Tullamarine, VIC, Australia). Macrophages were removed by treating the cells with anti-CD68 (Dako, Glostrup, Denmark) coated petri dishes for 20 min at 37 °C. Then, HNECs were seeded in collagen-IV coated T75 cell culture flasks (Corning Incorporated, Corning, NY, USA) and grown in Ex plus complete media (STEMCELL Technologies, Tullamarine, VIC, Australia). The seeded HNECs were incubated at 37 °C with a 95% humidity incubator supplied with 5% CO_2_ and inspected daily under light microscopy.

### 4.4. ALI Culture

Once the cells achieved 80–100% confluence, they were detached by treating with 0.05% trypsin (Thermo Scientific, Waltham, MA, USA) and resuspended in Ex plus complete media after centrifugation. The cell suspensions were then seeded onto collagen IV-coated apical chambers of Transwells (BD Biosciences, San Jose, CA, USA). Then, 500 μL Ex plus complete media was added to the basolateral chamber. The cells were cultured at 37 °C with 5% CO_2_ and given two to three days to settle, followed by the apical chamber medium being removed completely, and the basolateral chamber medium being changed to ALI complete media. The basolateral chamber medium was changed every two to three days. The cells were cultured for 17 to 21 days to allow for differentiation and tight junction formation.

### 4.5. Measurement of TEER

The TEER was measured using an EVOM2 epithelial volt-ohm meter (World Precision Instruments, Sarasota, FL, USA), using ohms per square centimetre (Ω/cm^2^). In brief, 100 μL and 500 μL of fresh ALI complete media were applied to the apical and basal chambers, respectively. The baseline TEER was measured. Only wells displaying baseline resistance readings greater than 700 Ω/cm^2^ were used for the experiments. The ALI complete media from the apical chamber was then removed, followed by adding 100 μL of ALI complete media medium containing the final concentration of mesalazine at different concentrations (0.5, 1, 5, 10, 20, and 50 mM). The negative control (ALI complete media) and positive control (10% Triton X-100, Sigma Aldrich, St. Louis, MO, USA) were tested alongside each other. The TEER was measured immediately after the treatment was applied and recorded as time point 0. The TEER was then measured every 15 min for the first hour and then in 30-min intervals for the remaining 6.5 h. Whilst taking the TEER measurements, a heating platform of 37 °C was used. The TEER values were normalised against the values obtained at time point 0.

### 4.6. Measurement of Paracellular Permeability Using Fluorescently Labelled Dextrans

The paracellular permeability was tested using 4-kDa fluorescein isothiocyanate-labelled (FITC) dextrans (Sigma Aldrich, St. Louis, MO, USA). After treating the cells with various concentrations of mesalazine for 7 h, the media of the basolateral chamber was replaced with fresh medium, the apical chamber medium was removed, and the cells were washed with phosphate buffer saline (PBS). Then, the apical chambers were filled with 3 mg/mL of FITC-Dextran and incubated for 2 h at 37 °C. The samples from the basolateral compartment were transferred to a clear bottom black 96-well plate (Corning-Costar Corp., Cambridge, UK). The fluorescence of the samples was then measured with a FLUOstar Optima 96-well fluorescence microplate reader (BMG Labtech, Ortenberg, Germany) at excitation and emission wavelengths of 485 nm and 520 nm. The experiment was repeated three times with cells from different donors.

### 4.7. Measuring Cytotoxicity with LDH Assay

Following the final TEER measurements, the medium was collected from the basal chambers of each sample and cytotoxicity was determined using the LDH release kit (Promega, Madison, WI, USA) according to the manufacturer’s instructions. Briefly, 50 µL of medium from each condition was transferred to a new plate, 50 µL of LDH reagent was added, and the plate was incubated for 30 min at room temperature in the dark. At 490 nm, absorbance was measured using a microplate reader (BMG Labtech, Ortenberg, Germany). Cells treated with ALI complete media and Triton X-100 served as negative and positive controls, respectively. The relative viability was determined by comparing the LDH levels of negative and positive controls. The experiment was conducted three times.

### 4.8. Measuring Cytotoxicity with MTT

HNECs and THP-1 cells were seeded at 1.2 × 10^6^ cells/well into 24 well tissue culture plates (Corning Incorporated, Kennebunk, ME, USA) containing 500 µL Ex Plus complete media (Stemcell Technologies, Vancouver, BC, Canada) and incubated at 37 °C with 5% CO_2_ until 80% confluence was achieved. The media was then removed, and the cells were washed with PBS. The cells were then treated with 500 µL Ex Plus complete media (Stemcell Technologies), with the final mesalazine concentration at 0.5, 1, 5, 10, 20, and 50 mM. Cells treated with media and 10% triton X-100 were used as a negative control and positive control respectively. Sterilised MiliQ water diluted media was used as a volume control and 0.25 mg/mL budesonide (0.5 mg/mL, AstraZeneca, Macquarie Park, NSW, Australia) as a positive treatment control. 50 µg/mL monosodium urate (MSU, 25 mg/mL, Sigma Aldrich, St. Louis, MO, USA) was used to stimulate the production of TNF-α. Plates were wrapped in foil and incubated at 37 °C and 5% CO_2_ for 24 h. The cells were then washed with 650 µL of PBS twice. Then, 200 µL of media and 50 µL MTT (5 mg/mL PBS) were added to each well and incubated for 4 h [38]. After that, the media and MTT were removed from the wells. Each well was then filled with 400 µL of Dimethylsulfoxide (DMSO) and 100 µL of glycine buffer (0.1 M glycine, 0.1 M sodium chloride (NaCl) adjusted to pH 10.5 with 1 M NaOH). After that, the plates were wrapped in aluminium foil and rocked on a Rocking Platform Mixer (Ratek Instruments, Mitcham, Australia) for 30 min. 100 µL was then taken and added in triplicate to new 96-well plates. A FLUOstar optima plate reader (BMG Labtech, Ortenberg, Germany) was used to read the absorbance of the plates at 570 nm.

### 4.9. Enzyme-Linked Immunosorbent Assay (ELISA) of IL-6

Samples from the basolateral chambers were collected after 7 h of mesalazine treatment to determine the IL-6 protein levels using an ELISA assay (BD Biosciences, Franklin Lakes, NJ, USA). Briefly, 96-well plates were coated with capture antibody in a 1:250 dilution to a final concentration of 2 µg/mL with coating buffer (0.1 M Sodium bicarbonate (NaHCO3)) overnight at 4 °C. The primary antibody was then removed, the plates were washed 2 times, and 200 µL of blocking buffer (PBS + 2% bovine serum albumin) was added to each well. 100 µL of samples were then added to wells and incubated at room temperature for 2 h. After washing, 100 µL of Biotin rat anti-human IL-6 antibody (BD Biosciences, Franklin Lakes, NJ, USA) diluted 1:1000 to 0.5 µg/mL in blocking buffer was added into each well. Samples were then incubated at room temperature for 30 min. Horseradish peroxidase (HRP)-conjugated streptavidin (Thermo Scientific, Walthman, MA, USA) was diluted 1:10,000 to 125 ng/mL in blocking buffer added to each well and incubated at room temperature for 30 min. 100 µL of tetramethylbenzidine (TMB) was then added and the sample was incubated at room temperature for 10 min. Stop solution was then added and absorbance was read at 450 nm with a FLUOstar OPTIMA plate reader (BMG Labtech, Ortenberg, Germany). An equal volume of sterilised MiliQ water was diluted into Ex Plus complete media (Stemcell Technologies) and served as volume control and treatment control of budesonide (0.25 mg/mL, AstraZeneca, Nacquarie Park, NSW, Australia) was also used.

### 4.10. Enzyme-Linked Immunosorbent Assay TNF-α—Cells Inflammation Model

HNEC and THP-1 cells were seeded into 24-well tissue culture plates as described above. The cells were stimulated with poly (I:C) (10 µg/mL in cell culture media), monosodium urate (MSU), volume control and a treatment control were used as described above. TNF-α was then measured using a TNF-α ELISA assay (Invitrogen, Frederick, MD, USA) at 3- and 6 h post-exposure. Briefly, an ELISA plate was coated with capture antibody (Purified Mouse anti-human TNF-α, BD Biosciences) and incubated overnight at 4 °C. Then, plates were blocked with a 100 µL blocking buffer. 100 µL samples were then added and incubated for 2 h at room temperature. The plates were then removed and washed three times. 100 µL of Biotin mouse anti-human TNF-α (BD Biosciences) was added to each well and incubated for 30 min at room temperature. HRP-conjugated streptavidin (Thermo Scientific, Walthman, MA, USA) was added to each well and incubated at room temperature for 30 min. 100 µL of TMB was then added after washing and incubated at room temperature for 10 min. Stop solution was then added and absorbance was read at 450 nm with a FLUOstar OPTIMA plate reader (BMG Labtech, Ortenberg, Germany).

### 4.11. Statistical Analysis

Statistical analysis of TEER, FITC dextran, LDH, MTT, IL-6, and TNF-α was assessed with an ANOVA followed by post hoc analysis, using Graph Pad Prism version 9.00 (GraphPad Software, La Jolla, CA, USA). Differences between groups were determined using a one-way analysis of variance (ANOVA). The significance was determined at a *p*-value < 0.05. The experiments were performed three times. The data is presented using mean ± standard error of the mean.

## 5. Conclusions

The result of this study indicates that mesalazine effectively reduces TNF-α, suggesting that it possesses anti-inflammatory characteristics. In addition, the excellent safety profile of mesalazine at dosages up to 20 mM suggests that it is not detrimental to HNECs and the mucosal barrier function at these concentrations. Overall, this could suggest that mesalazine is safe on nasoepithelial cells, and as part of a topical treatment for CRS, patients could be beneficial in lowering inflammation; however, further in vivo studies will have to be undertaken to confirm this anti-inflammatory effect in a sinonasal environment.

## Data Availability

Data is contained within the article and Supplementary Material.

## Supplementary Materials

The following supporting information can be downloaded at: https://www.mdpi.com/article/10.3390/ijms25052796/s1.
