# Supplementary material for: An In Vitro Study Evaluating the Safety of Mesalazine on Human Nasoepithelial Cells
Source: Int J Mol Sci. 2024 Feb 28;25(5):2796. doi: 10.3390/ijms25052796 (PMC10932137; doi:10.3390/ijms25052796)
Supplement: Supplementary file 1 [file ijms-25-02796-s001.zip › ijms-2879461-supplementary.pdf]

Supplementary Figure:

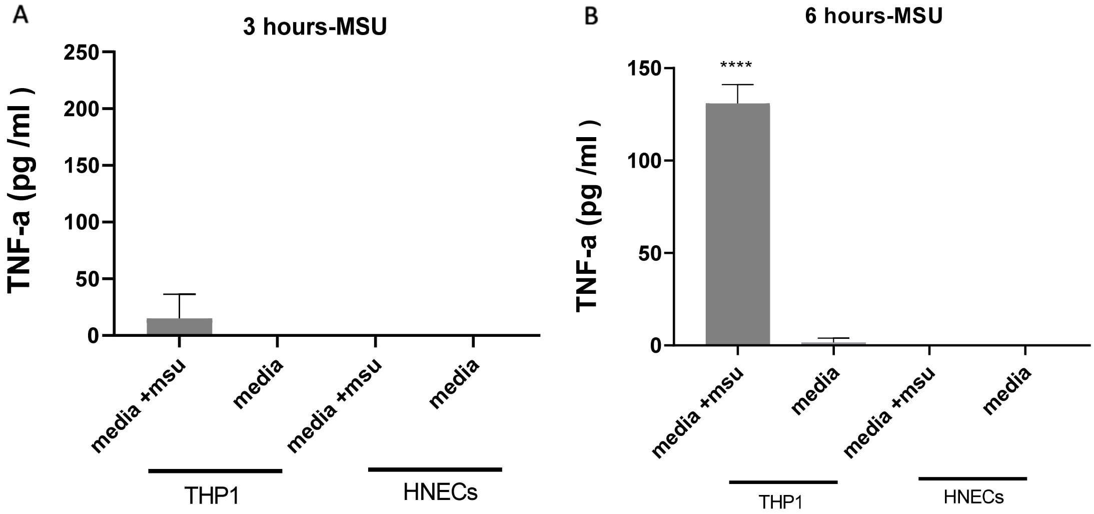

Supplementary figure S1: MSU can stimulate TNF- α in THP-1 cells, but not HNEC. (A) The production of TNF- α after stimulation with MSU for 3 hours for THP-1 cells and HNEC. (B) The production of TNF- α after stimulation with MSU for 6 hours for THP-1 cells and HNEC. Experiments were performed with three replicates. Negative control= Ex plus complete media.
